#  Integration of Lifestyle Medicine into the Medical Undergraduate Curriculum 

**DOI:** 10.30476/JAMP.2022.93916.1559

**Published:** 2022-04

**Authors:** VINU VIJ

**Affiliations:** 1 Department of Physiology, All India Institute of Medical Sciences, Nagpur, India

Dear Editor, 

One of the major challenges faced by the health care facilities globally is the significant shift of the global burden of disease from communicable diseases to non-communicable ones ( 1 ). The alarm on the rising rates of non-communicable diseases has been sounded by the public health, and epidemiological and clinical communities for at least the past forty years ( 2 ).

Lifestyle medicine provides an evidence-based solution to the problem of non-communicable diseases epidemic. However, the inclusion of lifestyle medicine in the medical undergraduate curriculum is minimal to non-existent. The medical school curriculum reform must include the mandatory training of all the medical undergraduate students in lifestyle medicine. This will resolve the inadequacies that exist in preparing physicians for the ever-growing challenge of non-communicable diseases they will have to treat and prevent ( 3 ). The resolution 959 was released in 2017 by the American Medical Association House of Delegates. It “supports policies and mechanisms that incentivize and/or provide funding for the inclusion of lifestyle medicine education and social determinants of health in undergraduate, graduate, and continuing medical education ( 3 , 4 ).”

Lifestyle medicine is the use of evidence-based lifestyle therapeutic intervention as a primary modality, delivered by trained and certified physicians, to prevent, treat, and often reverse chronic diseases. Lifestyle medicine intervention includes six pillars: a whole-food, plant predominant eating pattern, regular physical activity, restorative sleep, stress management, avoidance of risky substances, and positive social connection. As per the American College of Lifestyle Medicine, for clinicians to have sufficient knowledge and requisite skills to adequately serve the patients with chronic disease, medical educators must incorporate lifestyle medicine into undergraduate medical education, graduate medical education, and fellowship curriculum as well as continuing medical education and maintenance of certification. However, a huge void of adequate lifestyle medicine training exists across this entire continuum of medical education ( 4 ).

Medical school life can be a stressful period for students and may lead to the adoption of unhealthy lifestyle behaviours. Students must ideally be well equipped with the knowledge and skills to adopt healthy lifestyles to be able to flourish ( 5 ), but they lack such skills. In a study conducted in India to find out lifestyle disease risk behaviour in medical students, the authors concluded that unhealthy lifestyle disease behaviour is prevalent among them ( 6 ). Another study was conducted to determine the prevalence of healthy lifestyle behaviours in medical students, which was found to be quite low (31%) ( 5 ). This is largely because medical students do not have formal training in healthy lifestyle behaviours. Another study stated that only 14% of residents believed that they had the knowledge and training to provide nutrition counselling. The majority of residents were not aware of the guidelines for diagnosing obesity and they did not feel qualified to treat obese patients ( 4 ).

The American College of Lifestyle Medicine has convened the undergraduate medical education task force, which comprises of early adopting medical schools that have integrated lifestyle medicine across their curriculum through Lifestyle Medicine Interest Groups and lifestyle medicine elective courses. The task force has identified catalyzers for success as well as perceived challenges in implementing lifestyle medicine from a curriculum, institution, administration, faculty, and student stakeholder level ( 4 ). 

The policymakers and various stakeholders of all the countries should make an effort to collaborate with official bodies that have already implemented lifestyle medicine as a part of the medical curriculum and the public health system. This will be an important milestone in the medical curriculum reforms towards the actual realization of the ‘health for all’ goal of the World Health Organization.

## Conflict of Interest

None Declared.
